# Multiscale Modeling of Dendrimers and Their Interactions with Bilayers and Polyelectrolytes

**DOI:** 10.3390/molecules14010423

**Published:** 2009-01-19

**Authors:** Hwankyu Lee, Ronald G. Larson

**Affiliations:** 1 Laboratory of Computational Biology, National Heart, Lung, and Blood Institute, National Institutes of Health, Bethesda, MD, 20892, U.S.A.; 2 Departments of Chemical Engineering, Biomedical Engineering, Mechanical Engineering, and Macromolecular Science and Engineering Program, University of Michigan, Ann Arbor, MI, 48109, U.S.A.; E-mail: rlarson@umich.edu (R-G. L.)

**Keywords:** Simulation of dendrimer, Dendrimer-bilayer interaction, Dendrimer-induced pore formation, Dendrimer-DNA interaction.

## Abstract

Recent advances in molecular dynamics simulation methodologies and computational power have allowed accurate predictions of dendrimer size, shape, and interactions with bilayers and polyelectrolytes with modest computational effort. Atomistic and coarse-grained (CG) models show strong interactions of cationic dendrimers with lipid bilayers. The CG simulations with explicit lipid and water capture bilayer penetration and pore formation, showing that pore formation is enhanced at high dendrimer concentration, but suppressed at low temperature and high salt concentration, in agreement with experiments. Cationic linear polymers have also been simulated, but do not perforate membranes, evidently because by deforming into a pancake, the charges on a linear polymer achieve intimate contact with a single bilayer leaflet. The relatively rigid dendrimers, on the other hand, penetrate the bilayer, because only by interacting with both leaflets can they achieve a similar degree of contact between charged groups. Also, a “dendrimer-filled vesicle” structure for the dendrimer-membrane interaction is predicted by mesoscale thermodynamic simulations, in agreement with a picture derived from experimental observations. In simulations of complexes of dendrimer and polyelectrolyte, anionic linear chains wrap around the cationic dendrimer and penetrate inside it. Overall, these new results indicate that simulations can now provide predictions in excellent agreement with experimental observations, and provide atomic-scale insights into dendrimer structure and dynamics.

## 1. Introduction

**Figure 1:**
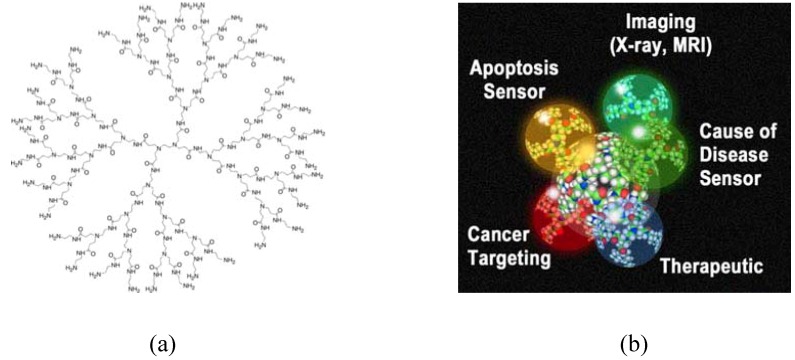
(a) Structure of generation 3 (G3) polyamidoamine (PAMAM) dendrimer. (b) Schematic of multifunctional PAMAM dendrimer. Images reproduced from http://nano.med.umich.edu

Dendrimers are branched polymers that consist of a central core, repeated building blocks, and many surface terminal groups [Figure 1(a)]. Due to their controlled mass, uniform structure, surface functionality, and good water solubility, they have been studied for many biomedical applications such as antitumor therapeutics and drug delivery [1,2,3,4,5]. For example, drugs, and sensing and imaging molecules can be attached to the dendrimer, and those complexes can be targeted to the specific cancer cell [Figure 1(b)]. To increase their targeting efficiency, interactions of dendrimers with lipid bilayers, DNA, and other molecules must be understood. Although experiments have provided vital information on the large-scale interactions between dendrimers and other molecules [6,7,8,9,10,11,12,13,14], many atomic-level questions that cannot be answered by experiments remain to be solved. Therefore, theoretical and computational modeling methods have been applied to investigate the atomic-scale insights into the interactions of dendrimers with other molecules. Initially, these consisted of Monte Carlo and Brownian dynamics simulations, but recent advances in computer speed and simulation methods have made it possible to study dendrimers and their interactions with explicit solvents and other molecules by atomistic and coarse-grained (CG) molecular dynamics (MD) simulations.

Lescanec and Muthukumar performed an off-lattice simulation of starburst molecules in three dimensions, which showed that the density is maximum at the center and decreases towards the surface terminal groups; this is called the “dense-core” model [15]. A similar result was obtained by Boris and Rubinstein using a modified equilibrium self-consistent mean field calculation [16]. More explicitly with atomic models, Naylor *et al*. performed the first MD simulations of generations 1 to 7 (G1-G7) polyamidoamine (PAMAM) dendrimers in a vacuum, showing the asymmetric shape of G1-G3, and the roughly spherical shape of G5-G7 [2]. Since then, many MD simulations of PAMAM dendrimers have been performed in vacuum, a review of which can be found in Ballauff and Likos [17] and Kandasamy *et al*. [18]. In this review, we will first (Section 2) briefly review the structure and dynamics of PAMAM dendrimers from recent atomistic MD simulations with explicit solvents. Next (Section 3), MD and mesoscale simulations of the interactions of PAMAM dendrimers with lipid bilayers will be reviewed. Lastly (Section 4), we will focus on simulations of the interactions between PAMAM dendrimers and polyelectrolyte such as DNA. 

## 2. Atomistic molecular dynamics simulations of dendrimers in explicit solvents

The Goddard group at Caltech performed the first atomistic MD simulations of G4 to G6 polyamidoamine (PAMAM) dendrimers in explicit water [19]. The simulation time was only 200 ps, but the radius of gyration (R_g_) calculated from the simulations agreed with those measured from experiments. Also, they found that the extent of ionization significantly affected the R_g_ of the dendrimer, which favorably compared with experimental values, where the extent of ionization was controlled by pH. They also showed that the thermodynamic and dynamic properties of water near a G5 PAMAM dendrimer differ significantly from those of bulk water [20]. In particular, protonation of amines on the dendrimer induced a decrease in water diffusion around the dendrimer, suggesting that binding of the dendrimer and other molecules at low pH may be much slower than that at high pH. Recently, they performed 20-40 ns-long MD simulations of a G8 PAMAM dendrimer in water, and showed that decreasing solvent pH from 10 to 4 significantly increased (by 13%) the dendrimer R_g_ [21], where the effect of pH was controlled by the extent of protonation for primary (terminal) and tertiary amines. This result disagreed with SANS experiments by Nisato *et al*., which Nisato *et al*. interpreted as measurements of isolated dense spherical objects [22]. Goddard and coworkers assumed that the disagreement resulted from a nonspherical dendrimer shape, penetration of water and ions into the core, and aggregation. They also found in their simulations significant back-folding of the primary amines, and penetration of water inside the dendrimer. 

In other studies, Han et al. simulated unprotonated G1 to G7 PAMAM dendrimers, showing that R_g_ scales with molecular weight to the power ~1/3 [23], as expected for dense spheroidal objects. Our group also performed MD simulations of charged and uncharged G5 dendrimers in both water and methanol, and showed that the R_g_ values match those obtained from experiments [24]. Hydrogen bonding of the dendrimer with itself or with water was analyzed, and its effects on dendrimer structure were investigated. The conformation of the dendrimer was found to be similar in water and methanol. Recently, Maiti and Messina simulated G1 to G7 PAMAM dendrimers with counterions and water, and showed a penetration of counterions into the dendrimer [25]. A higher local counterion concentration was observed within the larger dendrimers because of more back-folding of the dendrimer terminals at higher generations. They also found that the ζ-potential increases with dendrimer generation number, albeit slowly at higher generation number. The computed ζ-potential values compare favorably with those calculated from colloidal models (Monte Carlo simulations and Poisson-Boltzmann theory).

## 3. Simulations of dendrimers with lipid bilayers

If dendrimers are to deliver drugs and other useful molecules into cells, they must first interact with the cell membrane. Dendrimer-induced pore formation in lipid bilayers has been widely studied by atomic force microscopy and enzyme-leakage experiments [11], but to understand the mechanism atomic, coarse-grained, and mesoscale simulations have also been performed, which we review below.

### 3.1 Atomistic molecular dynamics simulations

Mecke *et al*. simulated G2-G5 PAMAM dendrimers in implicit solvent near a negatively charged plane modeled as a two-dimensional hexagonal array of beads [12], intended as a mimic of a mica surface or a bilayer. Their dendrimers, especially the charged ones, significantly flattened on the plane. They also showed that the bonded and nonbonded potential energy of the surface-deformed dendrimer increased exponentially with dendrimer generation. 

**Figure 2:**
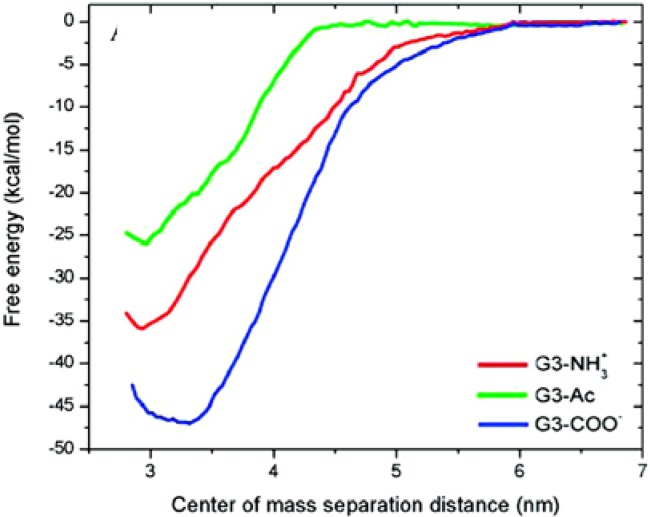
Potential of mean forces for G3 PAMAM dendrimers interacting with different surface charges as a function of center-of-mass separation distance between the dendrimer and lipid bilayer (energy per mole binding to the bilayers). Reprinted with permission from [26]. Copyright 2008 American Chemical Society.

Recently, Kelly *et al*. performed atomistic MD simulations of neutral, positively- and negatively-charged PAMAM G3 dendrimers (respectively, G3-Ac, G3-NH_3_^+^, and G3-COO^-^) with a dimyristoyl-phosphatidyl-choline (DMPC) bilayer with implicit solvent [26]. In Figure 2, free energies were calculated as functions of the distance between the dendrimer and lipid bilayer, showing that the total free energy released upon interaction with the bilayer is 26, 36, and 47 kcal/mol for G3-Ac, G3-NH_3_^+^, and G3-COO^-^, indicating that charged dendrimers more favorably interact with zwitterionic DMPC bilayers than do neutral dendrimers. Also, before contract (> 4.5 nm) between the dendrimer and lipid bilayer is made, G3-NH_3_^+ ^ and G3-COO^-^ show attractive interactions with the surface that are very similar to each other and higher than that of G3-Ac. These results indicate again that charged dendrimers more favorably interact with bilayers than do neutral dendrimers, and that the magnitude of dendrimer charge more significantly affects the interactions than does the sign of the charge. 

**Figure 3:**
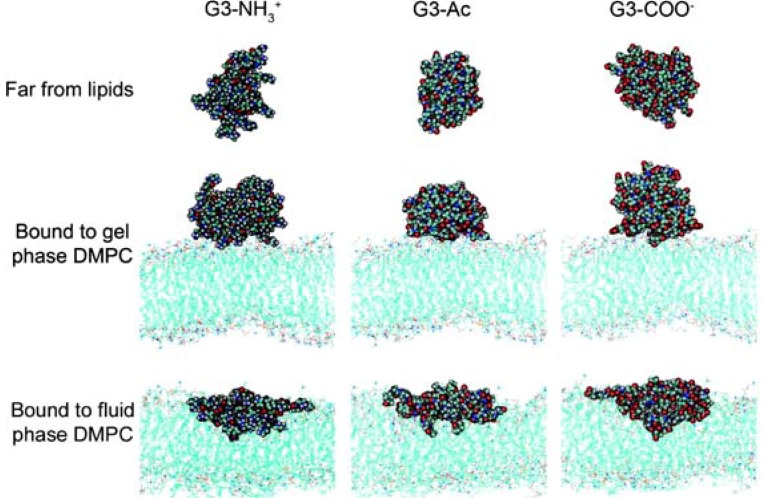
Snapshots of differently charged G3 dendrimers in fluid- and gel-phase DMPC bilayers. Reprinted with permission from [27]. Copyright 2008 American Chemical Society.

Kelly *et al*. also simulated differently charged G3 PAMAM dendrimers with different lipid phases (fluid vs. gel), showing that both charged and uncharged dendrimers retained a spherical shape on the gel-phase lipid bilayers, but flattened on the fluid-phase lipid bilayers (Figure 3) [27]. They also calculated the enthalpy release upon interaction of the dendrimer with the lipids, and showed that the stronger binding to the fluid-phase lipids was driven by the hydrophobic interactions between the inner dendrimer and the lipid tails. 

### 3.2 Coarse-grained molecular dynamics simulations

Due to limitations of system size and timescale, atomistic simulations of dendrimers with molecularly explicit lipid bilayers have only been carried out for small G3 dendrimers, which are of limited interest for applications, which normally use dendrimers at least as large as G5. Also, frequently only implicit solvent is used in atomistic simulations, because of the high simulation costs of including explicit water. To overcome these limitations, our group recently developed a coarse-grained (CG) model for charged and uncharged G3 to G7 dendrimers [28], which makes it possible to simulate multiple copies of these dendrimers with explicit, though coarse-grained, lipids and water for system sizes of up to 50 × 50 × 20 nm^3^. 

Our CG model for G3, G5, and G7 dendrimers was based on the MARTINI CG force field of Marrink *et al*. [29,30]. Although the MARTINI force field was parameterized for short-range electrostatic interactions by using a cutoff of 1.2 nm, we showed that long-range electrostatics using particle mesh Ewald (PME) summation does not affect the area per lipid and lateral diffusion coefficients of dendrimer-free DMPC bilayers [28]. Also, we found that incorporation of PME electrostatics was necessary to observe dendrimer-induced pore formation in lipid bilayers. In the MARTINI force field, there are about 12 different bead types differentiated by their charge and hydrophobicity, which we chose among to model nodes and branches in the interior parts of the dendrimer and the surface terminals. After choosing appropriate bead types for these moieties, equilibrium distances and angles with their force constants were adjusted to make the conformation of the dendrimers as close as possible to those from experiments. Since each CG bead represents four heavy atoms (not counting hydrogens), our CG G5 dendrimer has only 506 CG beads, while the original atomic G5 dendrimer has ~5,100 atoms. Similarly, the CG model lumps four water molecules into each “water” bead, greatly reducing the number of degrees of freedom in the simulation, without abandoning completely the use of explicit water molecules.

To validate this CG dendrimer model, we performed atomistic and CG MD simulations of charged and uncharged G5 dendrimers in water, and calculated their radii of gyration (R_g_) [24,28]. The differences in R_g_ between either CG or atomistic simulations and experiments were less than 10%. Uncharged G5 dendrimers showed lower values of R_g_ than charged G5, in agreement with the experimental results. These findings suggest that our CG dendrimer model successfully captures the effect of terminal-charge on dendrimer size. 

**Figure 4:**
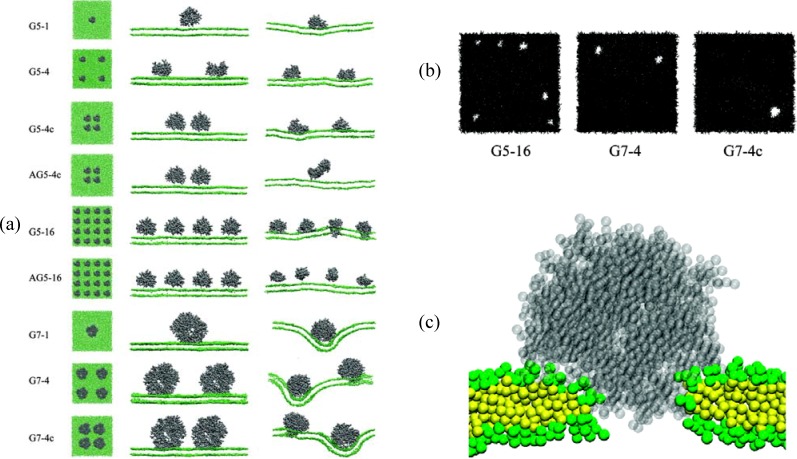
(a) Snapshots of the top view (left image) and the side view (middle image) at the beginning (0 ns) and the side view at the end (240 ns, right image) of simulations of multiple CG dendrimers with a DMPC bilayers. Gray dots represent dendrimers, and green dots represent headgroups of the DMPC bilayer. The explicit water molecules, DMPC tails, and ions are omitted for clarity. Note that side views show only one cross section of the system and cannot capture all dendrimers. (b) Top view of the DMPC bilayer at the end (240 ns) of simulations G5-16, G7-4, and G7-4c. (c) A snapshot of the dendrimer-induced pore in a DMPC bilayer at the end (240 ns) of the simulation G7-4c. Transparent gray dots represent a G7 dendrimer. Green and yellow dots represent head and tail groups of DMPC, respectively. Reprinted with permission from [31]. Copyright 2008 American Chemical Society.

We also performed CG MD simulations of the interactions of multiple copies of charged G5 and G7 and uncharged G5 dendrimers with DMPC bilayers in explicit water [31]. Snapshots from simulations with various dendrimer size, concentration, and terminal-charge density are shown in Figure 4(a). These dendrimer-bilayer systems are named “G5-1”, “G5-4”, “G5-4c”, “AG5-4c”, “G5-16”, “AG5-16”, “G7-1”, “G7-4”, and “G7-4”, where the first and second number describe the dendrimer generation and number of dendrimers, respectively. The initial “A” designates acetylation (uncharged), while the terminal “c” designates dendrimers that are initially clustered. When initially clustered together near the bilayer, neutral dendrimers aggregated, whereas cationic dendrimers dispersed, in agreement with the observations from atomic force microscopy by Mecke *et al*. [14]. Both charged G5 and G7 dendrimers induced significant bilayer deformation and pore formation on the positively curved portions of the bilayer [Figure 4(a) and Figure 4(b)], while neutral dendrimers did not form pores, indicating that higher dendrimer charge density induces more membrane curvature and facilitates pore formation. Pores were only observed to form in the systems with 16 G5 dendrimers and four G7 dendrimers (with no pore formation in the systems with four or fewer G5 dendrimers, or with only one G7 dendrimer), indicating a significant effect of dendrimer concentration on pore formation.

Figure 4(c) shows a snapshot of the dendrimer-induced pore at the end of the simulation of the system G7-4c. The DMPC head groups have become positioned between the dendrimer and DMPC tail regions. Interestingly, this pore structure approximates a toroidal pore, which has been also observed in simulations of other cationic peptides such as antimicrobial peptides. 

**Figure 5:**
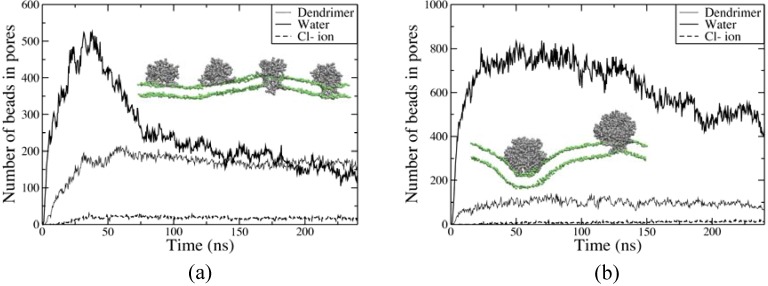
Numbers of dendrimer, water molecules, and ion beads in pores as a function of time, starting from a state with no performation of the bilayer for (a) G5-16 and (b) G7-4c. Reprinted with permission from [31]. Copyright 2008 American Chemical Society.

In these simulations, many dendrimer and water molecules were observed inside the pores, although the pores were much smaller than those observed in atomic force microscopy experiments. To compare the pore sizes induced by G5 and G7 dendrimers, the numbers of dendrimer and water beads inside each pore were computed. In Figure 5, more dendrimer and water molecules were found inside pores in the four-G7 dendrimer system than in the sixteen-G5 dendrimer system, although each system has the same number of total dendrimer charges. These results agree qualitatively with experimental observations of significant cytoplasmic-protein leakage induced by G7 dendrimers at concentrations as low as 10 nM, but by G5 dendrimers only at high concentration of 400-500 nM, suggesting that the dendrimer size and concentration significantly affect pore formation [8].

To investigate the effect of the polymer or dendrimer shape on pore formation, we also used the CG model to simulate a linear poly-L-lysine (PLL) polymer interacting with the lipid bilayer [32]. Experimental results have shown that even for PLL with a larger size (hydrodynamic radius) and higher charge density than a G5 dendrimer, the induced membrane permeability was almost the same, indicating that the dendrimer, with its spheroidal shape, may be more efficient, per unit mass, in increasing membrane permeability than is a polymer of linear shape [9]. Our simulations show that the G5 dendrimer modestly distorts in water to an ellipsoidal shape with aspect ratios of 1.2 and 1.4 respectively for *I_z_/I_y_* and *I_z_/I_x_*, where *I_z_, I_y_,* and *I_x_* are principal moments of inertia (ordered such that *I_z_ > I_y _* > *I_x_*), but that PLL128 (128 monomers in length) has aspect ratios of 1.1 and 7.3 respectively for *I_z_/I_y_* and *I_z_/I_x_*, indicating that PLL128 takes on a highly flattened pancake conformation. While the simulation with 16 G5 dendrimers carried the same number of charges as that with 16 PLL128 molecules, and both of these induced significant bilayer curvature, the system with 16 G5 dendrimers also induced pore formation, which did not occur in the system with 16 PLL128 molecules, indicating that the spheroidal shape of the former is more efficient in inducing membrane permeability. 

**Figure 6:**
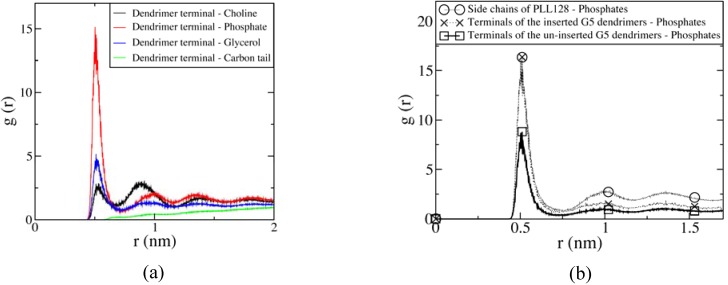
RDFs for (a) DMPC head and tail groups with respect to terminals of the pore-inducing G5 dendrimer, and for (b) DMCP phosphate head groups with respect to side chains of PLL128 and terminals of the inserted and un-inserted G5 dendrimers. Reprinted with permission from [32]. Copyright 2008 American Chemical Society.

The effects of linear and spheroidal shape may be explained in terms of charge interactions between dendrimer and lipid bilayer. In Figure 6, the radial distribution functions (RDF’s) show the effect of strong electrostatic interactions between the anionic phosphate groups of the bilayer and the cationic terminals of the dendrimer. Although PLL128 and the G5 dendrimer have the same number of positive charges per molecule, the RDF for the side chains of PLL128 has a much higher peak than does that of the terminals of the un-inserted dendrimers. This is expected, because all charged residues of linear polymers can interact with a single leaflet of the bilayer, but uninserted spheroidal dendrimers are not flexible enough to spread onto a single leaflet. Interestingly, the RDF for the terminal groups of the inserted dendrimers with the lipid phosphate is very close to that of the charged groups for PLL128, indicating that a dendrimer has to penetrate the bilayer to have favorable electrostatic interactions with head groups on the opposite leaflet, while the flexible linear polymers can, by flattening, gain all needed interactions with a single leaflet. These results suggest that a relatively rigid spheroidal shape is more efficient than a flexible linear shape in increasing membrane permeability.

In the simulations, charged dendrimer-induced pore formation was observed at 310 K, but not at 277 K, due to the condensed (gel) lipid bilayer of the latter [28], consistent with experimental observations [8]. At high salt concentration (~500 mM NaCl), charged G5 dendrimers did not insert into the bilayer, evidently because electrolyte weakens the electrostatic interactions between surface charges of dendrimers and the head groups of lipids. 

The good qualitative agreement with experimental findings indicates that the effect on pore formation of the size, shape, concentration, charge density of large nanoparticles as well as the effect of temperature and salt can be studied realistically through coarse-grained MD simulations. 

### 3.3 Mesoscale simulations

**Figure 7:**
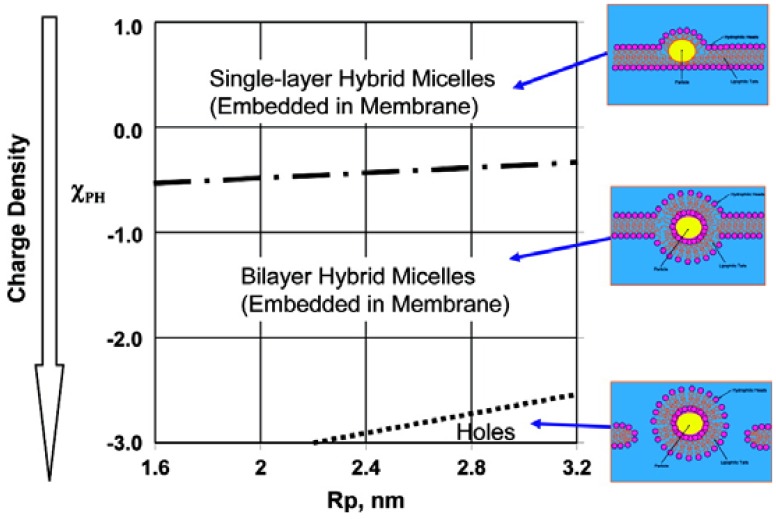
Phase diagram for the morphology of membrane-nanoparticle complex as a function of particle size (R_P_: two-dimensional particle radius) and surface charge (χ_PH_: Flory-Huggins interaction parameter between the particle and the headgroup). Reprinted with permission from [33]. Copyright 2007 American Chemical Society.

Ginzburg and Balijepalli carried out the combined Self-Consistent-Field/Density functional calculations to determine the morphology of the spherical nanoparticle-membrane interaction with the lowest free energy [33]. This morphology was investigated as a function of particle size and surface charge, yielding a phase diagram that shows that larger nanoparticle size and higher charge density induce insertion of the nanoparticle into membrane, leading to the formation of a nanoparticle-bilayer complex (Figure 7). Here, the simple spherical nanoparticle was modeled, but its behavior was considered to be very similar to that of the dendrimer because of their comparable particle shape. The predicted size and charge dependence agrees with observations from the dendrimer experiments, and with atomistic and coarse-grained MD simulations. Also, in the final nanoparticle-bilayer complex the lipid membrane encased the nanoparticle in a “spherical nanoparticle-filled vesicle”, which was proposed by Banaszak-Holl and co-workers as a means by which dendrimers remove material from the lipid membrane to create holes. In addition to this thermodynamic model, Smith *et al*. used Dissipative Particle Dynamics, showing that the particle is wrapped by the membrane, and then the raft’s interfacial energy drives a fission process, leading to detachment of the vesicle (wrapped particle) from the membrane [34]. These results indicate that mesoscale models may make it possible to map out the equilibrium structures of nanoparticle-membrane complexes as a function of nanoparticle properties.

## 4. Simulations of dendrimers with polyelectrolytes

Experimental studies have revealed a compact conformation of the DNA-dendrimer complex, and have explored its potential for drug and gene delivery applications [7,35,36]. In particular, DNA was observed to wrap around the dendrimer, which has motivated many simulation studies aimed at understanding the atomistic details of the process. Initially, Monte Carlo and Brownian dynamics simulations were mainly performed, and the electrostatic interactions were approximated by the Debye-Hückel potential (electrostatically screened Coulomb potential), where the effects of counterions and solvents were treated implicitly. However, the effects of counterions and solvents on the conformation of the dendrimer were shown to be significant [20,37], and hence atomistic and coarse-grained MD simulations have been recently performed with explicit counterions and solvents, where direct Coulomb potential and Ewald summation were used for electrostatics.

### 4.1 Monte Carlo and Brownian dynamics simulations

Welch and Muthukumar performed the first Monte Carlo simulations of the interactions between a cationic dendrimer (G4-G6) and an anionic polyelectrolyte. They showed that a dendrimer can encapsulate or interpenetrate a polyelectrolyte chain, or display a unique “chain-walking” phenomenon [38]. Here, “chain-walking” describes a process by which the dendrimer migrates from one end of the chain to the other via fluctuations in both chain and dendrimer conformations. They also predicted that the degree of polyelectrolyte encapsulation significantly depends on the salt concentration, size, and charge density of the dendrimer and the polyelectrolyte. Using Brownian dynamics simulations, Lyulin *et al*. studied the complex formed by a cationic dendrimer (G1-G4) with a sufficiently longer anionic polyelectrolyte chain (consisting of 24 to 90 negatively charged monomers) [39]. When the linear chain and the dendrimer had the same number of charges, the chain wrapped the dendrimer surface, and this decreased the radius of gyration of the dendrimer. For longer chains with more charges than the dendrimers, more chain monomers adsorbed onto the dendrimer than were necessary for dendrimer neutralization (i.e., there was over-compensation of charge). Recently, Lyulin *et al*. performed Brownian dynamics simulations, which showed very rare changes of lengths of the two tail regions of a sufficiently long polyelectrolyte attached to a dendrimer, which was described as a very slow “non-random-walk” process [40]. This result suggests that, contrary to the suggestion of Welch and Muthukumar [38], the motion of a dendrimer along with a polyelectrolyte cannot be described as a “chain-walking” phenomenon. 

### 4.2 Atomistic and coarse-grained molecular dynamics simulations

**Figure 8:**
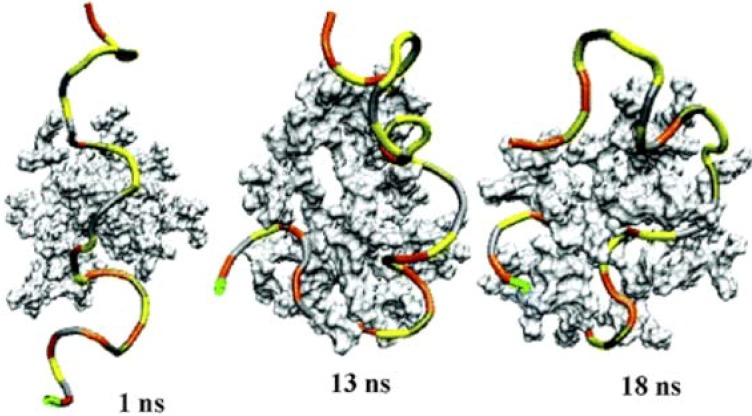
Snapshots every few ns of formation of an ssDNA-dendrimer complex. Reprinted with permission from [41]. Copyright 2006 American Chemical Society.

Maiti and Bagchi performed 20 ns-long atomistic MD simulations of G2-G4 dendrimer-ssDNA complexes in explicit water [41]. G2, G3, and G4 PAMAM dendrimers have 16, 32, and 64 protonated amines on their terminals, and their ssDNA had 37 negative charges. G2 and G3 did not contain enough surface charges to neutralize the ssDNA, and for these dendrimers only part of the ssDNA molecule coated the dendrimer to neutralize its charge, while some of the excess ssDNA coiled up near the dendrimer. While the work of Lyulin *et al*. described in the above paragraph showed that some charge over-compensation can occur when the linear chain has more charges than the dendrimer, the work of Maiti *et al*. evidently shows that under some circumstances the degree of over-compensation is very limited. However, a G4 dendrimer has enough positive charges (64) to neutralize the 37 charges on the ssDNA, and hence the ssDNA wrapped around the dendrimer and significantly penetrated inside of it, which swelled the dendrimer (Figure 8). The free energy was shown to be minimized for the dendrimer-ssDNA complexed state. The base sequence of the DNA significantly influenced the stability of the complex because of a competition between binding enthalpy and bending rigidity of ssDNA. Since the latter is sequence dependent, this phenomenon might be of use in the analysis of DNA base sequences. dsDNA is much stiffer than ssDNA, but the simulations showed that it too could be significantly bent through interactions with a G3 dendrimer. 

To overcome limitations of system size and timescale in atomistic MD simulations, Lyulin *et al*. performed coarse-grained simulations of a dendrimer-polyelectrolyte complex [42]. Their dendrimer had 48 positive charges on its surface, and the linear polyelectrolyte had 10 (for the monovalent chain) or 20 (for the divalent chain) negative charges. A compact dendrimer-polyelectrolyte complex was observed, and stronger electrostatic interaction of the divalent chain led to a decreased dendrimer size, and an increased dehydration of the chain. These results suggest that charged dendrimers are able to encapsulate guest chains and screen them from the surrounding solvent. 

To summarize the above theoretical work on the interactions of linear polyelectrolytes with oppositely charged dendrimers, if N_C_ (number of charged monomers on linear chain) ≤ N_T_ (number of dendrimer terminals), the dendrimer “encapsulates” the polyelectrolyte, and the dendrimer-polyelectrolyte complex becomes more compact than the free dendrimer. If N_C_ > N_T_, the dendrimer still encapsulates part of the polyelectrolyte, but the excess charges of the chain are repelled, and the dendrimer/polymer complex becomes larger than the free dendrimer. 

## 5. Conclusions

Much useful information about the structure and dynamics of dendrimers has been gained, initially using Monte Carlo and Brownian dynamics simulations, and atomistic molecular dynamics (MD) simulations with vacuum, and, as computer speed has advanced, increasingly using atomistic and coarse-grained (CG) MD simulations with explicit water. Atomistic MD simulations in explicit water over a timescale of tens of nanoseconds have yielded dendrimer radii of gyration (R_g_) in good agreement with experiments. R_g_ was found to scale with molecular mass to the expected power of ~1/3, and the effects of solvent type and pH on the size of the dendrimer have also been determined, and are in agreement with experimental observations. Water near the dendrimer shows slower diffusion than bulk water. The ζ-potential was found by simulation to slowly increase with dendrimer generation, in agreement with a colloidal model for the dendrimer analyzed by Monte Carlo simulations and Poisson-Boltzmann theory. 

The successful simulations of the dendrimer structure have provided a basis for extending the simulations to the interactions of dendrimers with other molecules. Because of their possible use as antitumor agents or DNA transfection agents, most experimental studies have focused on the interactions of dendrimers with lipid bilayers, and with DNA. Atomistic MD simulations in implicit water showed that a G3 PAMAM dendrimer flattens against a fluid-phase lipid bilayer. Free energies calculated as a function of the distance between the dendrimer and lipid bilayer showed that charged dendrimers more favorably interact with bilayers than do neutral dendrimers. CG MD simulations of larger G5 and G7 dendrimers with explicit lipids in explicit CG water in systems as large as 50 × 50 × 20 nm^3^ can now be performed economically. In such simulations, increased bilayer curvature and pore formation result from higher dendrimer generation, and higher dendrimer concentration or charge density. Dendrimer-induced pores in lipid bilayers approximate toroidal pores, which are proposed structures of pores induced by antimicrobial peptides. Simulations with linear poly-L-lysine (PLL) showed bilayer curvature, but no pore formation. Although the flexible linear polymer PLL has more intimate electrostatic interactions with the head groups of the bilayer, rigid spheroidal dendrimers induce more membrane curvature and pore formation because they cannot spread onto a single leaflet, and so must penetrate the bilayer to have favorable electrostatic interactions with head groups on the opposite leaflet. These results indicate that the rather rigid spheroidal shape of dendrimers may make them more efficient in increasing membrane permeability than are flexible-chain linear polymers. Even with dendrimers, pore formation was not observed at low temperature where the membrane enters the gel phase, nor at high salt concentration, presumably due to weakened electrostatic interactions. Besides atomistic and CG MD simulations, density functional calculations have been used to describe the thermodynamics of particle-membrane interactions. These have shown the effects of the particle size and charge on structure and have predicted a “dendrimer-filled vesicle” structure that had earlier been hypothesized as a mechanism by which dendrimers remove material from lipid membranes.

The formation of complexes of dendrimer and polyelectrolyte (such as DNA) has been also simulated. Monte Carlo and Brown dynamics simulations show strong charge interactions between the cationic dendrimer terminals and anionic polyelectrolyte chains. More precisely, atomistic and CG MD simulations show that despite their rigidity, strong electrostatic interactions cause linear DNA chains to wrap around the dendrimer and penetrate inside it, leading to formation of a compact complex.

Multiscale simulations have successfully matched experimentally measured properties, and provide atomic-scale insights into structure and dynamics of dendrimers and their interactions with other molecules. This information from simulations can help in the rational design of nanoparticle size, shape, or surface properties for applications in nanomedicine. 
